# Supplementary material for: Advice on Regulating Body Mass in Wrestling from the Most Cited Combat Sport Literature—A Systematic Review
Source: J Funct Morphol Kinesiol. 2024 Dec 9;9(4):264. doi: 10.3390/jfmk9040264 (PMC11678334; doi:10.3390/jfmk9040264)
Supplement: Supplementary file 1 [file jfmk-09-00264-s001.zip › Suplemenatry exclusion criteria.pdf]

Suplementary file S2

All 60 papers

Exclusion criteria: not about combat sport, not about weight regulation, duplicate papars.

Web of science			
Weight loss Combat sport			
Authors			
Franchini, E; Brito, CJ; Artioli, GG	Weight loss in combat sports: physiological, psychological and performance effects	2012	Paper 1
Gómez-Cabello, A; Ara, I; González-Agüero, A; Casajús, JA; Vicente-Rodríguaz, G	Effects of Training on Bone Mass in Older Adults	2012	Not about combat sport
Artioli, GG; Gualano, B; Franchini, E; Scagliusi, FB; Takesian, M; Fuchs, M; Lancha, AH	Prevalence, Magnitude, and Methods of Rapid Weight Loss among Judo Competitors	2010	Paper 2
Brito, CJ; Roas, AFCM; Brito, ISS; Marins, JCB; Córdova, C; Franchini, E	Methods of Body-Mass Reduction by Combat Sport Athletes	2012	Paper 3
Pocecco, E; Ruedl, G; Stankovic, N; Sterkowicz, S; Del Vecchio, FB; Gutiérrez-García, C; Rousseau, R; Wolf, M; Kopp, M; Miarka, B; Menz, V; Krüsmann, P; Calmet, M; Malliaropoulos, N; Burtscher, M	Injuries in judo: a systematic literature review including suggestions for prevention	2013	Not about weight loss
Rapid weight loss Combat sport			
Authors			
Franchini, E; Brito, CJ; Artioli, GG	Weight loss in combat sports: physiological, psychological and performance effects	2012	Duplicate paper
Artioli, GG; Gualano, B; Franchini, E; Scagliusi, FB; Takesian, M; Fuchs, M; Lancha, AH	Prevalence, Magnitude, and Methods of Rapid Weight Loss among Judo Competitors	2010	Duplicate paper
Brito, CJ; Roas, AFCM; Brito, ISS; Marins, JCB; Córdova, C; Franchini, E	Methods of Body-Mass Reduction by Combat Sport Athletes	2012	Duplicate paper
Artioli, GG; Iglesias, RT; Franchini, E; Gualano, B; Kashiwagura, DB; Solis, MY; Benatti, FB; Fuchs, M; Lancha, AH	Rapid weight loss followed by recovery time does not affect judo-related performance	2010	Paper 4
Chaabène, H; Tabben, M; Mkaouer, B; Franchini, E; Negra, Y; Hammami, M; Amara, S; Chaabène, RB; Hachana, Y	Amateur Boxing: Physical and Physiological Attributes	2015	Not about weight regulation
Weight cycling Combat sport			
Authors			
Franchini, E; Brito, CJ; Artioli, GG	Weight loss in combat sports: physiological, psychological and performance effects	2012	Duplicate paper
Pocecco, E; Ruedl, G; Stankovic, N; Sterkowicz, S; Del Vecchio, FB; Gutiérrez-García, C; Rousseau, R; Wolf, M; Kopp, M; Miarka, B; Menz, V; Krüsmann, P;	Injuries in judo: a systematic literature review including suggestions for prevention	2013	Not about weight regulation

Calmet, M; Malliaropoulos, N; Burtscher, M			
Miller, WC	How effective are traditional dietary and exercise interventions for weight loss?	1999	Not about combat sport
Frost, DM; Cronin, J; Newton, RU	A Biomechanical Evaluation of Resistance Fundamental Concepts for Training and Sports Performance	2010	Not about weight regulation
Owens, JG; Blair, JA; Patzkowski, JC; Blanck, RV; Hsu, JR	Return to Running and Sports Participation After Limb Salvage	2011	Not about combat sport
Weight control Combat sport			
Authors			
Gómez-Cabello, A; Ara, I; González-Agüero, A; Casajús, JA; Vicente-Rodríguaz, G	Effects of Training on Bone Mass in Older Adults	2012	Not about combat sport
Artioli, GG; Gualano, B; Franchini, E; Scagliusi, FB; Takesian, M; Fuchs, M; Lancha, AH	Prevalence, Magnitude, and Methods of Rapid Weight Loss among Judo Competitors	2010	Duplicate paper
Artioli, GG; Iglesias, RT; Franchini, E; Gualano, B; Kashiwagura, DB; Solis, MY; Benatti, FB; Fuchs, M; Lancha, AH	Rapid weight loss followed by recovery time does not affect judo-related performance	2010	Duplicate paper
Bledsoe, GH; Hsu, EB; Grabowski, JG; Brill, JD; Li, GH	Incidence of injury in professional Mixed Martial Arts competitions	2006	Not about weight regulation
Miller, WC	How effective are traditional dietary and exercise interventions for weight loss?	1999	Not about combat sport
Weight management Combat sport			
Authors			
Artioli, GG; Gualano, B; Franchini, E; Scagliusi, FB; Takesian, M; Fuchs, M; Lancha, AH	Prevalence, Magnitude, and Methods of Rapid Weight Loss among Judo Competitors	2010	Duplicate paper
Pocecco, E; Ruedl, G; Stankovic, N; Sterkowicz, S; Del Vecchio, FB; Gutiérrez-García, C; Rousseau, R; Wolf, M; Kopp, M; Miarka, B; Menz, V; Krüsmann, P; Calmet, M; Malliaropoulos, N; Burtscher, M	Injuries in judo: a systematic literature review including suggestions for prevention	2013	Not about weight regulation
Burke, LM; Close, GL; Lundy, B; Mooses, M; Morton, JP; Tenforde, AS	Relative Energy Deficiency in Sport in Male Athletes: A Commentary on Its Presentation Among Selected Groups of Male Athletes	2018	Not about combat sport
Artioli, GG; Franchini, E; Nicastro, H; Sterkowicz, S; Solis, MY; Lancha, AH	The need of a weight management control program in judo: a proposal based on the successful case of wrestling	2010	Paper 5
Reale, R; Slater, G; Burke, LM	Weight management Practices of Australian Olympic Combat Sport Athletes	2018	Paper 6
Rapid weight gain			
Authors	Article Title		
Artioli, GG; Saunders, B; Iglesias, RT; Franchini, E	It is Time to Ban Rapid Weight Loss from Combat Sports	2016	Paper 7
Pettersson, S; Ekström, MP; Berg, CM	Practices of Weight Regulation Among Elite Athletes in Combat Sports: A Matter of Mental Advantage?	2013	Paper 8
Reale, R; Slater, G; Burke, LM	Acute-Weight-Loss Strategies for Combat Sports and Applications to Olympic Success	2017	Paper 9
Reale, R; Slater, G; Burke, LM	Weight management Practices of Australian Olympic Combat Sport Athletes	2018	Duplicate paper

Matthews, JJ; Nicholas, C	Extreme Rapid Weight Loss and Rapid Weight Gain Observed in UK Mixed Martial Arts Athletes Preparing for Competition	2017	Paper 10
Scopus			
Rapid weight loss			
Artioli G.G., Iglesias R.T., Franchini E., Gualano B., Kashiwagura D.B., Solis M.Y., Benatti F.B., Fuchs M., Junior A.H.L.	Rapid weight loss followed by recovery time does not affect judo-related performance	(2010),	Duplicate paper
Artioli G.G., Saunders B., Iglesias R.T., Franchini E.	It is Time to Ban Rapid Weight Loss from Combat Sports	(2016),	Duplicate paper
Franchini E., Brito C.J., Artioli G.G.	Weight loss in combat sports: Physiological, psychological and performance effects	(2012),	Duplicate paper
Brito C.J., Roas A.F.C.M., Brito I.S.S., Marins J.C.B., Córdova C., Franchini E.	Methods of body-mass reduction by combat sport athletes	(2012),	Duplicate paper
Pettersson S., Ekström M.P., Berg C.M.	Practices of weight regulation among elite athletes in combat sports: A matter of mental advantage?	(2013),	Duplicate paper
Weight loss Combat sport			
Artioli G.G., Iglesias R.T., Franchini E., Gualano B., Kashiwagura D.B., Solis M.Y., Benatti F.B., Fuchs M., Junior A.H.L.	Rapid weight loss followed by recovery time does not affect judo-related performance	(2010),	Duplicate paper
Franchini E., Brito C.J., Artioli G.G.	Weight loss in combat sports: Physiological, psychological and performance effects	(2012),	Duplicate paper
Brito C.J., Roas A.F.C.M., Brito I.S.S., Marins J.C.B., Córdova C., Franchini E.	Methods of body-mass reduction by combat sport athletes	(2012),	Duplicate paper
Pettersson S., Ekström M.P., Berg C.M.	Practices of weight regulation among elite athletes in combat sports: A matter of mental advantage?	(2013),	Duplicate paper
Carroll S., Dudfield M.	What is the relationship between exercise and metabolic abnormalities? A review of the metabolic syndrome	(2004),	Not about combat sport
Weight cycling Combat sport			
Mendes S.H., Tritto A.C., Guilherme J.P.L.P., Solis M.Y., Vieira D.E., Franchini E., Lancha A.H., Jr., Artioli G.G.	Effect of rapid weight loss on Performance in combat sport male athletes: Does adaptation to chronic weight cycling play a role?	(2013),	Paper 11
Franchini E., Brito C.J., Artioli G.G.	Weight loss in combat sports: Physiological, psychological and performance effects	(2012),	Duplicate paper
Owens J.G., Blair J.A., Patzkowski J.C., Blanck R.V., Hsu J.R.	Return to running and sports participation after limb salvage	(2011),	Not about combat sport
Reale R., Slater G., Burke L.M.	Weight management practices of australian olympic combat sport athletes	(2018),	Duplicate paper
Matthews J.J., Stanhope E.N., Godwin M.S., Holmes M.E.J., Artioli G.G.	The magnitude of rapid weight loss and rapid weight gain in combat sport athletes preparing for competition: A systematic review	(2019),	Duplicate paper
Weight control Combat sport			
Artioli G.G., Iglesias R.T., Franchini E., Gualano B., Kashiwagura D.B., Solis M.Y., Benatti F.B., Fuchs M., Junior A.H.L.	Rapid weight loss followed by recovery time does not affect judo-related performance	(2010),	Duplicate paper
Burke L.M., Close G.L., Lundy B., Mooses M., Morton J.P., Tenforde A.S.	Relative energy deficiency in sport in male athletes: A commentary on its presentation among selected groups of male athletes	(2018),	Not about combat sport

Costarelli V., Stamou D.	Emotional Intelligence, Body Image and Disordered Eating Attitudes in Combat Sport Athletes	(2009),	Paper 12
Ngai K.M., Levy F., Hsu E.B.	Injury trends in sanctioned mixed martial arts competition: A 5-year review from 2002 to 2007	(2008),	Not about weight regulation
Bledsoe G.H., Hsu E.B., Grabowski J.G., Brill J.D., Li G.	Incidence of injury in professional mixed martial arts competitions	(2006),	Not about weight regulation
Weight menagement Combat sport			
Burke L.M., Close G.L., Lundy B., Mooses M., Morton J.P., Tenforde A.S.	Relative energy deficiency in sport in male athletes: A commentary on its presentation among selected groups of male athletes	(2018),	Not about combat sport
Matthews J.J., Nicholas C.	Extreme rapid weight loss and rapid weight gain observed in UK mixed martial arts athletes preparing for competition	(2017),	Duplicate paper
Petraglia A.L., Maroon J.C., Bailes J.E.	From the field of play to the field of combat: A review of the pharmacological management of concussion	(2012),	Not about weight regulation
Coswig V.S., Miarka B., Pires D.A., Da Silva L.M., Bartel C., Del Vecchio F.B.	Weight regain, but not weight loss, is related to competitive success in real-life mixed martial arts competition	(2019),	Paper 13
Reale R., Slater G., Burke L.M.	Weight management practices of australian olympic combat sport athletes	(2018),	Duplicate paper
Rapid weight gain Combat sport			
Artioli G.G., Saunders B., Iglesias R.T., Franchini E.	It is Time to Ban Rapid Weight Loss from Combat Sports	(2016),	Duplicate paper
Reale R., Cox G.R., Slater G., Burke L.M.	Regain in body mass after weigh-in is linked to success in real life judo competition	(2016),	Duplicate paper
Matthews J.J., Nicholas C.	Extreme rapid weight loss and rapid weight gain observed in UK mixed martial arts athletes preparing for competition	(2017),	Duplicate paper
Pettersson S., Ekström M.P., Berg C.M.	Practices of weight regulation among elite athletes in combat sports: A matter of mental advantage?	(2013),	Duplicate paper
Reale R., Slater G., Burke L.M.	Acute-weight-loss strategies for combat sports and applications to olympic success	(2017),	Duplicate paper
